# Noise-robust optimization of quantum machine learning models for polymer properties using a simulator and validated on the IonQ quantum computer

**DOI:** 10.1038/s41598-022-22940-4

**Published:** 2022-11-08

**Authors:** Yuki Ishiyama, Ryutaro Nagai, Shunsuke Mieda, Yuki Takei, Yuichiro Minato, Yutaka Natsume

**Affiliations:** 1 Platform Laboratory for Science and Technology, Asahi Kasei Corporation, Shizuoka, Japan; 2 Informatics Initiative, Asahi Kasei Corporation, Tokyo, Japan; 3 Blueqat Inc., Tokyo, Japan; 4 Present Address: Informatics Initiative, Asahi Kasei Corporation, Tokyo, Japan

**Keywords:** Cheminformatics, Quantum information

## Abstract

Quantum machine learning for predicting the physical properties of polymer materials based on the molecular descriptors of monomers was investigated. Under the stochastic variation of the expected predicted values obtained from quantum circuits due to finite sampling, the methods proposed in previous works did not make sufficient progress in optimizing the parameters. To enable parameter optimization despite the presence of stochastic variations in the expected values, quantum circuits that improve prediction accuracy without increasing the number of parameters and parameter optimization methods that are robust to stochastic variations in the expected predicted values, were investigated. The multi-scale entanglement renormalization ansatz circuit improved the prediction accuracy without increasing the number of parameters. The stochastic gradient descent method using the parameter-shift rule for gradient calculation was shown to be robust to sampling variability in the expected value. Finally, the quantum machine learning model was trained on an actual ion-trap quantum computer. At each optimization step, the coefficient of determination $R^{2}$ improved equally on the actual machine and simulator, indicating that our findings enable the training of quantum circuits on the actual quantum computer to the same extent as on the simulator.

## Introduction

Materials informatics^1^, which is a collaboration between materials and information sciences, has been attracting attention as a highly-efficient method to search for materials with innovative performance. For example, in the quantitative structure–property relationship^2^, chemical structures are quantified based on their substructures and chemical properties (referred to as descriptors)^3^, and a regression model between the descriptors and target properties is constructed. By inverse analysis of the regression model, materials with ideal properties can be screened based on their chemical structure^4^. Recently, several databases such as PubChem^5^ and Materials Project^6^, containing more than tens of thousands of material properties, have been developed. Therefore, innovations in computer technology that can more effectively utilize the accumulated data are desired.

In recent years, quantum computers have attracted attention as devices that have the potential to outperform conventional computer technology^7,8^. However, the development of fault-tolerant quantum computers could require a decade of research. Currently, quantum computing using noisy intermediate-scale quantum (NISQ) devices^9,10^, which are medium-scale quantum computers composed of hundreds of noisy qubits, is being studied. Although NISQ devices can only implement algorithms on a limited quantum circuit scale owing to noise limitations, research is underway for their practical applications in the fields of chemistry^11–14^ and finance^15,16^. Quantum chemical calculation is an application of quantum computers in the field of chemistry, and the variational quantum eigensolver (VQE)^17^ is a typical algorithm used in this field. Recently, Gao et al. reported the calculation of excited states of organic electroluminescent (EL) light-emitting materials on an actual quantum computer^18^. They examined the effect of the noise inherent in real quantum computers on quantum chemical calculations, which has been a problem in the past. By using error mitigation based on quantum tomography, they succeeded in obtaining calculated values that correlated well with experimental values.

Another application of quantum computers in chemistry is materials informatics using quantum machine learning^19^. An example of machine learning using a quantum computer is linear regression using D-Wave's quantum annealing system by Date and Potok^20^. They reported that, compared with the classical approach, the quantum approach can train models up to 2.8 times faster on large datasets, and the regression error is comparable to that of the classical approach. However, reports of machine learning with quantum annealing systems predominantly utilize linear regression, and furthermore, annealing systems are considered not well-suited for representing continuous numbers owing to the precision issues of quantum processing units (QPUs)^21^. Therefore, to further exploit the power of quantum machine learning, it is also important to consider quantum machine learning using gate-based quantum computers, which allow for more flexible model construction.

A representative quantum machine learning algorithm that can be run on gate-based NISQ quantum computers is the quantum circuit learning (QCL) algorithm^22–24^. The QCL algorithm consists of two circuits: encoding and variational. The encoding circuit converts the classical feature *x* into a high-dimensional quantum-enhanced feature space. Subsequent variational circuits act on the feature space generated by the encoding circuits and learn the relationship between the feature space and a target variable *y,* using trainable parameters contained in the variational circuit. The predicted value of the target variable is calculated from the expected value of the Pauli Z measurement to a few qubits in the quantum circuit. Therefore, it should be noted that the predicted values are stochastic because they are calculated based on the expected values obtained from a finite number of shots (measurements). QCL is a hybrid quantum–classical algorithm^25^ wherein the relationship between the explanatory variable $x$ and the target variable *y* is represented by a parameterized circuit on a quantum computer $\left( {y = f_{\theta } \left( x \right)} \right)$, whereas the optimization of the parameters is performed on a classical computer.

An example of research using QCL in the field of chemistry is the prediction of molecular toxicity from molecular descriptors (Quantitative Structure–Activity Relationship) by Suzuki and Katouda^26^. They investigated the effect of the encoding structure and variational circuits on the prediction accuracy using a quantum computer simulator. A QCL model with high prediction accuracy for toxicity prediction tasks was proposed. This suggests that the QCL is a useful approach in the field of chemistry. However, to the best of our knowledge, whereas the application of quantum machine learning to regression has been studied using a quantum computer simulator, the application of the regression task on a real quantum computer has not yet been sufficiently studied. Therefore, further research is needed on the effects of stochastic fluctuations in predictions and noise specific to quantum computers on the training of the model, which can be a problem in real quantum computers.

In this study, we investigated the QCL in a regression task to predict the physical properties of a homopolymer from the corresponding monomer structure (quantitative structure–property relationship). Assuming conditions similar to those of an actual quantum computer, we investigated the effect of stochastic variation in the expected predicted values obtained from a quantum circuit on parameter optimization. To enable parameter optimization even under the noise that can occur in real machines, we examined quantum circuits that improve prediction accuracy without increasing the number of trainable parameters, and parameter optimization methods that are robust to the expected stochastic variations. Finally, we performed parameter optimization of the QCL using a real quantum computer (IonQ's quantum computer) to verify whether parameter optimization is possible under the noise generated by a real computer.

## Methods

### Data

For quantum machine learning, we used 86 monomer-polymer property datasets generated by the Synthia module of the Materials Studio 2019 software, which is a tool that calculates the properties of the corresponding polymer from the structure of the monomer. For the explanatory variable *x*, we used 10 monomer features related to structural information of monomers (unit length, molecular weight, connectivity indices^27^, etc.) calculated using Synthia. For the target variable *y*, the glass transition temperature (*T*_g_) of the homopolymer calculated using Synthia was used. To reduce computational costs, principal component analysis (PCA)^28^ was applied to the explanatory variables, and the first through fourth principal components were used as explanatory variables (cumulative contribution ratio > 99%).

### Quantum circuit learning (QCL) model

Two types of quantum circuits were considered: quantum circuits proposed in previous studies and multi-scale entanglement renormalization ansatz (MERA) circuits^29^. The details of these quantum circuits are described in the Results and Discussion section. The predicted value of the target variable was calculated from the expectation value obtained by applying the Pauli Z operator to the last qubit in the quantum circuit proposed in a previous study, and to the third qubit in MERA. The mean square error was used as the objective function to optimize the training parameters of the quantum circuit. To evaluate the performance of the model, we used the coefficients of determination $R^{2}$ and $Q^{2}$, which are expressed by the following equations:

$$R^{2} = 1 - \frac{{\mathop \sum \nolimits_{i = 1}^{N} \left( {y - y_{{{\text{calc }},i}} } \right)^{2} }}{{\mathop \sum \nolimits_{i = 1}^{N} \left( {y - \overline{y}} \right)^{2} }}$$

$$Q^{2} = 1 - \frac{{\mathop \sum \nolimits_{i = 1}^{N} \left( {y - y_{{{\text{pred }},i}} } \right)^{2} }}{{\mathop \sum \nolimits_{i = 1}^{N} \left( {y - \overline{y}} \right)^{2} }}$$

where *N* is the total number of samples,$y$ is the actual value of the objective variable, $\overline{y}$ is the average of $y$,$y_{{{\text{calc}}}}$ is the calculated value of $y$ (predicted value of *y* for training data in the model trained on all samples), and $y_{{\text{pred }}}$ is the predicted value of $y$ in tenfold cross-validation. Because of the limitations of the quantum circuit, the following preprocessing was performed on the explanatory variable *x* and the objective variable *y*. First, because *x* was converted into an angle on the Bloch sphere, a min–max normalization was applied so that the range of *x* was − π to π. Furthermore, because the Z expectation value of the quantum circuit ranges from − 1 to 1, *y* was min–max normalized to minimum and maximum value of − 1 and 1, respectively, and then transformed by the tanh function to minimum and maximum values of − 0.76 and 0.76, respectively (= tanh 1). This is because when the range of *y* in the training data is normalized from − 1 to 1, it becomes impossible to predict new samples beyond the range of *y* in the training data.

### Implementation

Python was used for the implementation. Blueqat^30^, a gate-based quantum computer simulator, was used for the simulation of QCL. The Blueqat SDK was used to run and simulate the IonQ quantum computer for comparison with an actual machine. Scikit-learn^31^ was used for data preprocessing. The Nelder–Mead^32^ method and Adam optimizer^33^, from the SciPy^34^ library, were used to optimize the quantum machine learning parameters.

## Results and discussion

**Figure 1:**
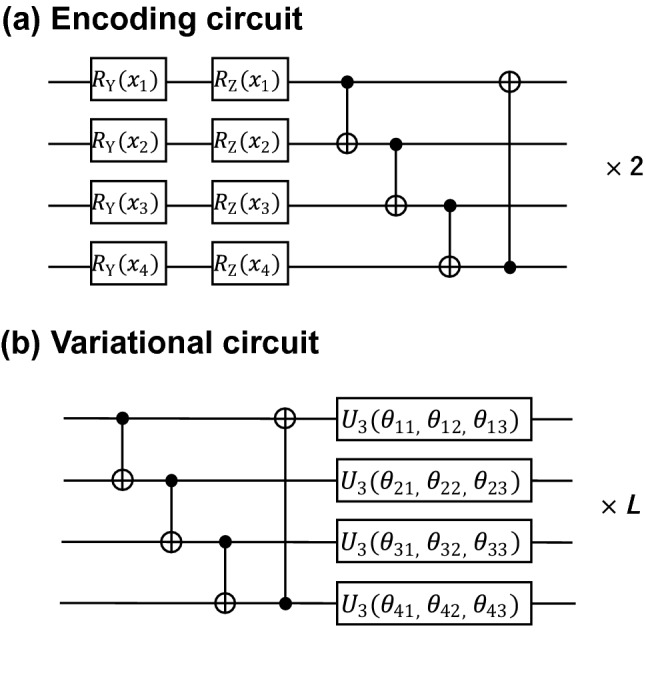
(**a**) Structure of an encoding circuit that converts the classical explanatory variable *x* into a quantum-enhanced, high-dimensional feature space. (**b**) Structure of a variational circuit that learns the relationship between the feature space and target variable. An arbitrary rotation gate $U_{3}$ contains trainable parameters. The variational circuit can be repeated *L* times (which is a hyperparameter). The total number of trainable parameters is 12 × *L*.

To investigate quantum machine learning for polymer property datasets, we first examined a previously proposed quantum circuit (hereinafter referred to as the original circuit)^26^, shown in Fig. 1. The encoder circuit (Fig. 1a) consists of one-qubit rotation gates $\left( {R_{{\text{Y}}} ,R_{{\text{Z}}} } \right)$ and controlled NOT (CNOT) gates. The one-qubit rotation gates convert the explanatory variable $x$ into a quantum state and the subsequent CNOT gate allows the quantum state to be entangled, allowing for a flexible representation of the feature space. As in the previous study, the one-qubit rotation gate and subsequent CNOT gate operations were repeated twice. The variational circuit (Fig. 1b) consists of a CNOT gate and an arbitrary rotation gate for each qubit, and it was repeated *L* times. As each arbitrary rotation gate contains three trainable parameters, the total number of trainable parameters per layer is 12 (= 3 × 4).

**Figure 2:**
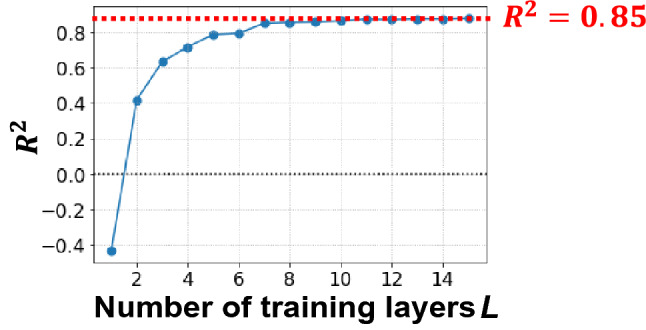
Change in the coefficient of determination $R^{2}$ as the number of training layers *L* is increased. The expected predicted value obtained from the quantum circuit is calculated directly from the state vector held by the simulator.

To verify the performance of the model under ideal conditions where there is no variation in the expected value calculation in sampling, the expected predicted value obtained from the quantum circuit was calculated directly from the state vector held by the simulator. The Nelder–Mead method^35^ was used to optimize the parameters, as in previous studies. Figure 2 shows the change in the coefficient of determination $R^{2}$ as the number of training layers *L* increased. At *L* = 1, $R^{2} < 0$, but at *L* = 2, $R^{2}$ significantly improved to $R^{2} = 0.42$, and then increased as the number of layers *L* increased, reaching a ceiling at $R^{2} = 0.85$ around *L* = 8.

**Figure 3:**
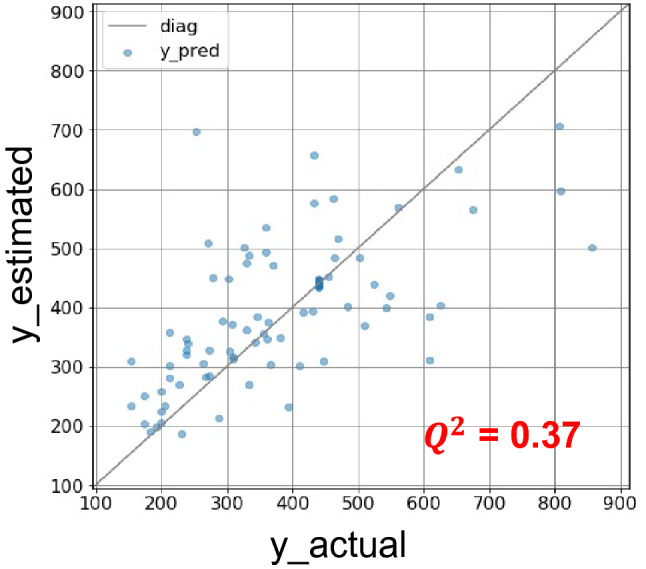
Actual *y* versus estimated *y* plot in the tenfold cross-validation with *L* = 8 model. The expected predicted value obtained from the quantum circuit is calculated directly from the state vector held by the simulator.

To verify the prediction performance (generalization performance) of the model on new data, tenfold cross-validation was conducted on the *L* = 8 model. The coefficient of determination for the predictions in the tenfold cross-validation was $Q^{2} = 0.37$. Figure 3 shows the actual versus estimated *y* values plot. Although there were some samples that missed the prediction by a large margin, the prediction results were generally distributed on the diagonal. This suggests that even simple quantum circuits can learn the trends in the complex relationship between the monomer structure and polymer properties.

When training a model on a real quantum computer, the expected predicted value obtained from the quantum circuit cannot be calculated directly from the state vector; it must be calculated from a sampling of stochastically fluctuating measurements. With finite sampling, the expected value may vary from the true value, and parameter optimization may be adversely affected. Therefore, we next examined the effect of the variation of the expected predicted value associated with a finite number of samplings on the optimization of the parameters.

**Figure 4:**
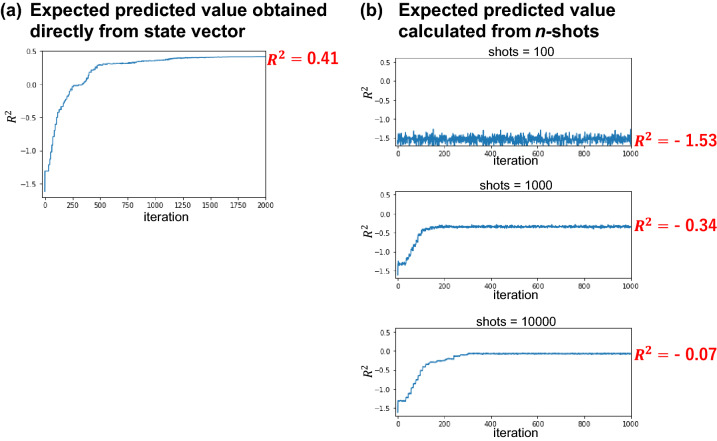
Comparison of the learning curves when the expected predicted value is obtained directly from the state vector (left) and when it is calculated from n-shots sampling (*n* = 100, 1000, 10,000) (right). Although the learning curve improves as the number of shots is increased, $R^{2}$ still converges at a negative value even when *n* = 10,000, which may be due to the stochastic variation in the expected predicted value.

Figure 4b shows the learning curves for parameter optimization of the original quantum circuit (*L* = 2) when the number of shots (samplings) to obtain the expected predicted value for each sample is 100, 1000, and 10,000. For comparison, the learning curve for the case where the expected value is obtained directly from the state vector is also shown (Fig. 4a). Note that because the expected value calculation is performed for each sample, the total number of samplings per optimization step, that is, the total number of samplings to evaluate the loss function based on the training parameters in a certain optimization step, is determined by (number of samples) × (shots per sample). The number of samples was 86.

Figure 4b shows that as the number of shots increases, the noise in the learning curve decreases and the final $R^{2}$ value improves. This can be attributed to the fact that the probabilistic variation of the expected value decreases as the number of shots increases. However, even for n = 10,000 shots, $R^{2}$ converged at a negative value. With a small number of shots, the direction of parameter optimization using the Nelder–Mead method may not be determined owing to the effect of stochastic variation of the expected predicted values. Increasing the number of shots further from 10,000 would improve the learning curve. However, in actual machines, an increase in the number of shots not only leads to an increase in the calculation time, but also in the machine running cost. Therefore, it is necessary to consider a quantum circuit that can be trained with fewer learning parameters, which facilitates parameter optimization, and an optimization method that can optimize parameters robustly to stochastic variations in expected values, even with a small number of shots.

**Figure 5:**
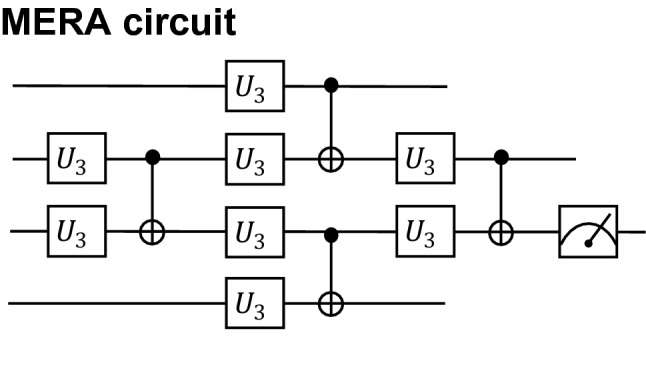
Structure of the MERA circuit. Each arbitrary rotation gate *U*_3_ contains three trainable parameters. Therefore, the total number of trainable parameters is 24, which is the same as that of the original circuit with *L* = 2. The predicted value of the target variable was calculated from the expected value obtained by applying the Pauli Z operator to the third qubit.

To improve the accuracy without increasing the number of trainable parameters, we considered the MERA circuit structure shown in Fig. 5. MERA is a structure that approximates the quantum state of a many-body system and can represent certain quantum states efficiently using only a few parameters^36^. Focusing on this fact, we verified whether it is possible to efficiently train a model with fewer parameters by using MERA as a variational circuit in QCL. The trainable parameters are the rotation angles at each arbitrary rotation gate $U_{3}$, and the total number of parameters is 24 for the four-qubit circuit used in this study. The encoding circuit is the same as that shown in Fig. 1a. First, to verify the performance of the model under ideal conditions that do not include the effects of probabilistic expected value variations, the expected predicted value obtained from the quantum circuit was calculated directly from the state vector held by the simulator.

**Figure 6:**
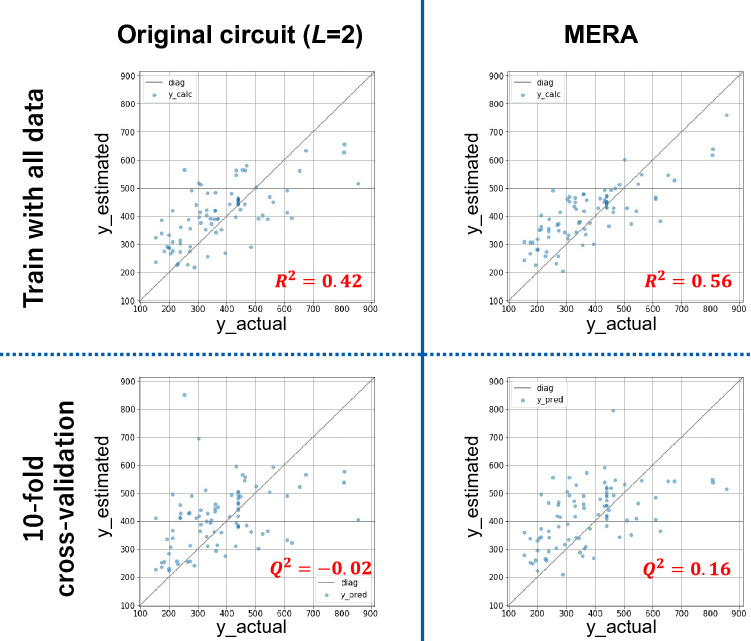
Comparison of actual *y* versus estimated *y* plots when using the original circuit (*L* = 2) or the MERA circuit as the variational circuit. The left column shows the plots for the original circuit (*L* = 2), and the right column shows those for the MERA circuit. The top row shows the estimation of *y* trained on all the data, and the bottom row shows the results predicted through the tenfold cross-validation. The expected predicted value obtained from the quantum circuit was calculated directly from the state vector held by the simulator.

Figure 6 shows a comparison between the actual *y* and estimated *y* plots for the original circuit (*L* = 2), and the MERA circuit with the same number of trainable parameters (24). The top row of Fig. 6 shows the prediction results of the training data after training the model on all data, and the bottom row shows the prediction results of the tenfold cross-validation. The performance of the model is $R^{2} = 0.42$ and $Q^{2} = - 0.02$ for the original circuit (*L* = 2) compared to $R^{2} = 0.56$ and $Q^{2} = 0.16$ for the MERA type, indicating that the model performance improves even with the same number of trainable parameters. Furthermore, in other datasets, MERA also tended to have higher $R^{2}$ and $Q^{2}$ values than the original circuit under the same number of trainable parameters (see Supplementary Table S1 and Fig. S1 online). Based on the trend of higher $R^{2}$ and $Q^{2}$ values for MERA, we conclude that MERA appears to learn more efficiently with fewer parameters than the original circuit in quantum machine learning.

Next, to enable parameter optimization that is robust to variations in the expected predicted value obtained from the quantum circuit even with a small number of shots, the parameter optimization method was changed from the Nelder–Mead method to the proposed method^37^. Previous studies have shown that the proposed method can optimize quantum circuits with a small number of shots, but no comparison has been made between the proposed method and other optimization methods. Therefore, it is unclear whether the proposed method improves the robustness of optimization against stochastic variation in expected values, even when optimization using the Nelder–Mead method is difficult owing to stochastic variations in expected values, as in the present case. In addition, as the previous study was conducted on a quantum computer simulator, it is necessary to verify whether the proposed method can optimize the quantum circuit even on a noisy real-world quantum computer. The details of the change from the Nelder–Mead method to the proposed method are as follows:

1. Stochastic gradient descent (SGD) with the Adam optimizer^33^ was adopted as the parameter optimization method.
2. The parameter-shift rule^23^ was used to calculate the gradient in the SGD.

Although the Nelder–Mead method has the advantage of not using the gradient information of the objective function, it tends to increase the number of optimization steps. When the number of training parameters is large, such as in neural networks, optimization by the Nelder–Mead method is often difficult, and the method using the gradient of the objective function is often used. Therefore, in this case, we changed the optimization method of the parameters to the gradient descent method. Furthermore, when optimization is performed using all the samples, the expected value must be calculated for all samples. Therefore, the total number of shots per optimization step increased in proportion to the number of samples. To reduce the total number of shots per optimization step, we adopted the SGD method, which updates the parameters using only one randomly selected sample per optimization step.　The total number of shots can be suppressed using SGD in QCL. Furthermore, owing to the randomness of the SGD, convergence to the local minimum can be suppressed even if the number of parameters is large.

The gradient of the objective function was calculated using the parameter-shift rule, which is a method for calculating the gradient of the output of a parameterized quantum circuit (i.e., the expectation of an observable). The gradient of the expected value of the quantum circuit observable $B$, for the circuit parameters $\theta$ of the quantum gate $U\left( \theta \right) = {\text{ exp}}\left( { - {\text{i}}\theta P} \right) \left( {P \in \left\{ {X, Y , Z} \right\}} \right)$ is as follows:

$$\frac{{{\text{d}}B\left( \theta \right)}}{{{\text{d}}\theta }} = \frac{1}{2}\left( {B\left( {\theta + \frac{\pi }{2}} \right) - B\left( {\theta - \frac{\pi }{2}} \right) } \right)$$

In other words, the gradient is calculated from the expected value of observable $B$ for a quantum circuit in which the parameter $\theta$ is increased or decreased by $\frac{\pi }{2}$. Compared with the calculation of the gradient using the finite difference method, the parameter-shift rule can theoretically obtain the exact derivative and is robust to errors^38^.

**Figure 7:**
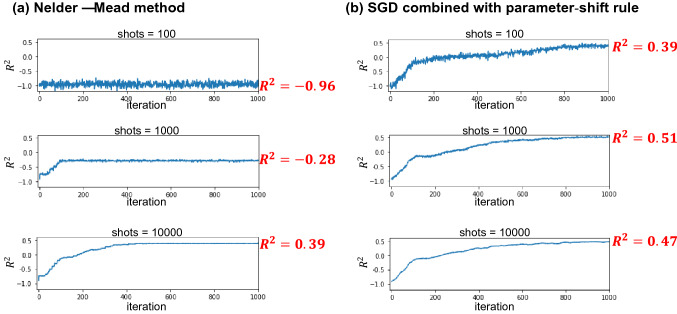
Comparison of learning curves for different parameter optimization methods when optimizing a quantum circuits using MERA as a variational circuit from the same initial values. (**a**) Nelder–Mead method. (**b**) SGD combined with parameter-shift rule. We considered 100, 1000, and 10,000 as the number of shots n to calculate each expected value. Combining the SGD method with the parameter-shift rule greatly improves the dependence of the learning curve on the number of shots.

Figure 7 compares the dependence of the learning curve on the number of shots for quantum circuits using MERA as the variational circuit when optimized from the same initial values using the conventional Nelder–Mead method and the SGD method with parameter- shift rule, respectively. Whereas the Nelder–Mead method results in a low $R^{2}$ value when the number of shots is 100 or 1000, the present optimization method can even optimize the parameters even with 100 shots, and the dependence of the learning curve on the number of shots is greatly reduced.

To train with the Nelder–Mead method, approximately 10,000 shots are required for each expected value calculation. Because the calculation of the objective function requires the expected value of all samples (86 in this case), the total number of shots for each optimization step is 860,000 (= 10,000 × number of samples). By contrast, in the SGD combined with the parameter-shift rule, optimization is possible even with 100 shots. The total number of shots per optimization step is 4,900 (= 100 + 2 × 100 × 24)—note that for a single stochastically selected sample, two expectation calculations are needed to compute the gradient of each trainable parameter (24 in total) using the parameter-shift rule, in addition to calculating the predicted value of *y*. Thus, by changing the optimization method, we were able to reduce the number of shots per optimization step to approximately $\frac{1}{180}$, which is a more reasonable value for optimization on an actual quantum computer.

We also confirmed that the SGD method improves the shot number dependence of the learning curve not only in MERA but also in the original circuit (L = 2), suggesting that the improvement of the shot number dependence of the learning curve by the SGD method is not a case specific to the MERA circuit (see Supplementary Fig. S2 online). Note that even when the original circuit (L = 2) and MERA are optimized with the SGD method, MERA still has a higher $R^{2}$. Compared to the conventional Nelder–Mead method, the SGD using the parameter-shift rule enables robust optimization against stochastic variation of the expected value and allows optimization with a small number of shots.

Finally, by using MERA as a variational circuit and optimizing it using SGD with a parameter-shift rule, we verified that the model can be trained on a real quantum computer that includes noise not accounted for by the simulator. For the quantum computer, we used IonQ's quantum computer^39^ (hereinafter referred to as IonQ), which is an ion-trap quantum computer. IonQ has a low error rate for each qubit, and all 11 qubits are fully coupled, which makes it possible to implement a wide range of quantum algorithms. Owing to machine cost and computation time, we did not verify the entire learning process on the actual machine, but rather investigated whether the model could outperform $R^{2} = 0$ in parameter optimization, that is, whether it could outperform a model that always outputs the average value of the target variable.

**Figure 8:**
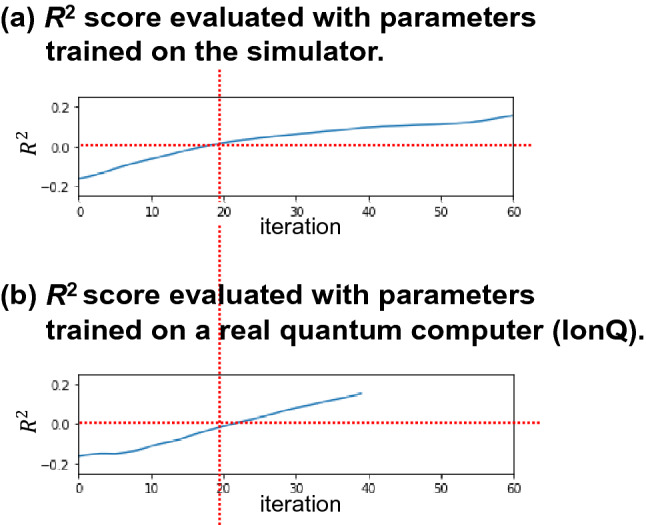
Comparison of the learning curves obtained through the (**a**) simulator and (**b**) a real quantum computer (IonQ). The number of shots for each expected value calculation is 100. Even on a noisy real quantum computer, the parameters are optimized just as in the simulator.

To reduce the machine cost, the initial values of the parameters optimized by IonQ were not random, but those optimized beforehand in 60 steps with 1,000 shots from random initial values in the simulator. Figure 8 shows the learning curve of the 40-step optimization using the IonQ quantum computer and simulator from the same initial values, with 100 shots for each expected value calculation. The evaluation of $R^{2}$ for each step to draw the learning curve, which is not necessary for the training process of the model, was calculated directly from the state vector held by the simulator using parameters optimized on an actual quantum computer or simulator. This is to eliminate uncertainty due to finite sampling in the calculation of $R^{2}$ and to properly compare the optimization process of the parameters themselves. Figure 8 shows that $R^{2}$ improves in the actual quantum computer to the same extent as in the simulator, and $R^{2}$ exceeds zero after approximately 20 steps. The SGD method and the parameter-shift rule enable robust optimization, even in the presence of variations in the expected value owing to the sampling of finite solutions and the noise inherent in a real quantum computer. Note that in Fig. 7b, approximately 200 steps are required to reach $R^{2}$ ~ 0, whereas in Fig. 8b, only 60 (initial steps before IonQ input) $+$ 20 steps are required, due to the different random number of initial state. This indicates that the learning speed of this model (the number of steps required for training) may largely depend on the initial state.

## Conclusion

In this study, we constructed a QCL model for a polymer physical property dataset and investigated the effect of noise generated in a real quantum computer on model learning.

First, the quantum circuits proposed in previous studies were evaluated. It was found that the training of quantum circuits does not progress when the probabilistic variation of the expected predicted values obtained from quantum circuits is considered. Therefore, the construction of variational circuits that improve prediction accuracy was examined without increasing the number of trainable parameters, and parameter optimization methods that are robust to stochastic variations in the expected values were studied.

The MERA-type circuit improved the prediction accuracy over the original circuit containing the same number of trainable parameters. In addition, the parameter optimization method was changed from the conventional Nelder–Mead method to the SGD method, where the gradient is calculated using the parameter-shift rule. This greatly improved the dependence of the learning curve on the number of shots to calculate the expected predicted value, allowing the model to be trained with a small number of shots, which is realistic for a real quantum computer.

Finally, we examined the training of a quantum circuit on an ion-trap quantum computer (IonQ). As the optimization progressed, the coefficient of determination of the model trained with IonQ improved to the same level as that of the simulator. By combining the SGD method and the parameter-shift rule, it was verified that the QCL model can be trained robustly with a small number of shots, even on a noisy real quantum computer. Although many challenges must be overcome to achieve practical accuracy in QCL, we believe that the results of this research, wherein quantum machine learning was performed on an actual quantum computer, are of great value as basic research on quantum machine learning.

## Supplementary Information

Supplementary Information.

## Data Availability

The datasets generated during and/or analysed during the current study are available from the corresponding author on reasonable request.
