# Supplementary material for: Noise-robust optimization of quantum machine learning models for polymer properties using a simulator and validated on the IonQ quantum computer
Source: Sci Rep. 2022 Nov 8;12:19003. doi: 10.1038/s41598-022-22940-4 (PMC9643424; doi:10.1038/s41598-022-22940-4)

**Supplementary Information**

Noise-robust optimization of quantum machine learning models for polymer properties using a simulator and validated on the IonQ quantum computer

**Yuki Ishiyama^1*,2^, Ryutaro Nagai^3^, Shunsuke Mieda^1,2^, Yuki Takei^1,2^, Yuichiro Minato^3^, Yutaka Natsume^1,2^**

^1^ Platform Laboratory for Science and Technology, Asahi Kasei Corporation, Shizuoka, Japan

^2^ Informatics Initiative, Asahi Kasei Corporation, Tokyo, Japan

^3^ Blueqat Inc., Tokyo, Japan

*ishiyama.yc@om.asahi-kasei.co.jp

**Comparison of prediction accuracy of original (L=2) and MERA circuits on other chemistry datasets**

We compared the performance of the original (L=2) and MERA circuits on a chemical dataset that differed from the dataset used in the main text (**Dataset 1**). The explanatory variable *x* and the objective variable *y* for each dataset were as follows.

| **Dataset 2**: | Same *x* as in Dataset 1 but different *y* (refractive index). *y* values were generated using Synthia as in Dataset 1. As in Dataset 1, the total number of samples was 86, but one sample with a large *y* outlier was removed and 85 samples were used. |
| --- | --- |
| **Dataset 3**: | Same *x* as in Dataset 1, but different *y* (bulk modulus). *y* values were generated using Synthia as in Dataset 1. As in Dataset 1, the total number of samples was 86, but one sample with a large *y* outlier was removed and 85 samples were used. |
| **Dataset 4:** | Different *x* and *y* from Dataset 1. The dataset used was that used to predict aqueous solubility from molecular descriptors in Ref. [Delaney, J. S. ESOL: Estimating aqueous solubility directly from molecular structure. *J Chem Inf Comput Sci* **44**, 1000–1005 (2004)]. For *x*, we used the same four variables as in the reference: molecular lipophilicity (logP), molecular weight, number of rotatable bonds, and aromatic proportion. *x* was calculated using the Python library RDkit. As *y*, we used experimental solubility values obtained from the reference. The total number of samples was 1143. For further details on the dataset, please refer to the reference. |

Table S1. Summary of $R^{2}$ and $Q^{2}$ values for the original (L=2) and MERA circuits for each dataset. MERA circuits tend to have higher $R^{2}$ and $Q^{2}$ values. For k-fold cross-validation to calculate the $Q^{2}$ value, k=10 was used for Datasets 1–3, and k=5 for Dataset 4 to reduce the computational cost.

|  | Number of samples | Original (L=2)  $R^{2}$ | MERA  $R^{2}$ | Original (L=2)  $Q^{2}$ | MERA  $Q^{2}$ |
| --- | --- | --- | --- | --- | --- |
| Dataset 1 | 86 | 0.42 | **0.56** | - 0.02 | **0.16** |
| Dataset 2 | 85 | 0.54 | **0.61** | 0.05 | **0.30** |
| Dataset 3 | 85 | 0.65 | **0.72** | **0.51** | 0.50 |
| Dataset 4 | 1143 | 0.25 | **0.52** | 0.25 | **0.51** |

Figure S1. Comparison of plots of actual *y* versus estimated *y* in cross-validation for the original (L = 2) and MERA circuits in each dataset. For k-fold cross-validation to calculate the $Q^{2}$ value, k=10 was used for Datasets 1–3, and k=5 for Dataset 4 to reduce computational cost.


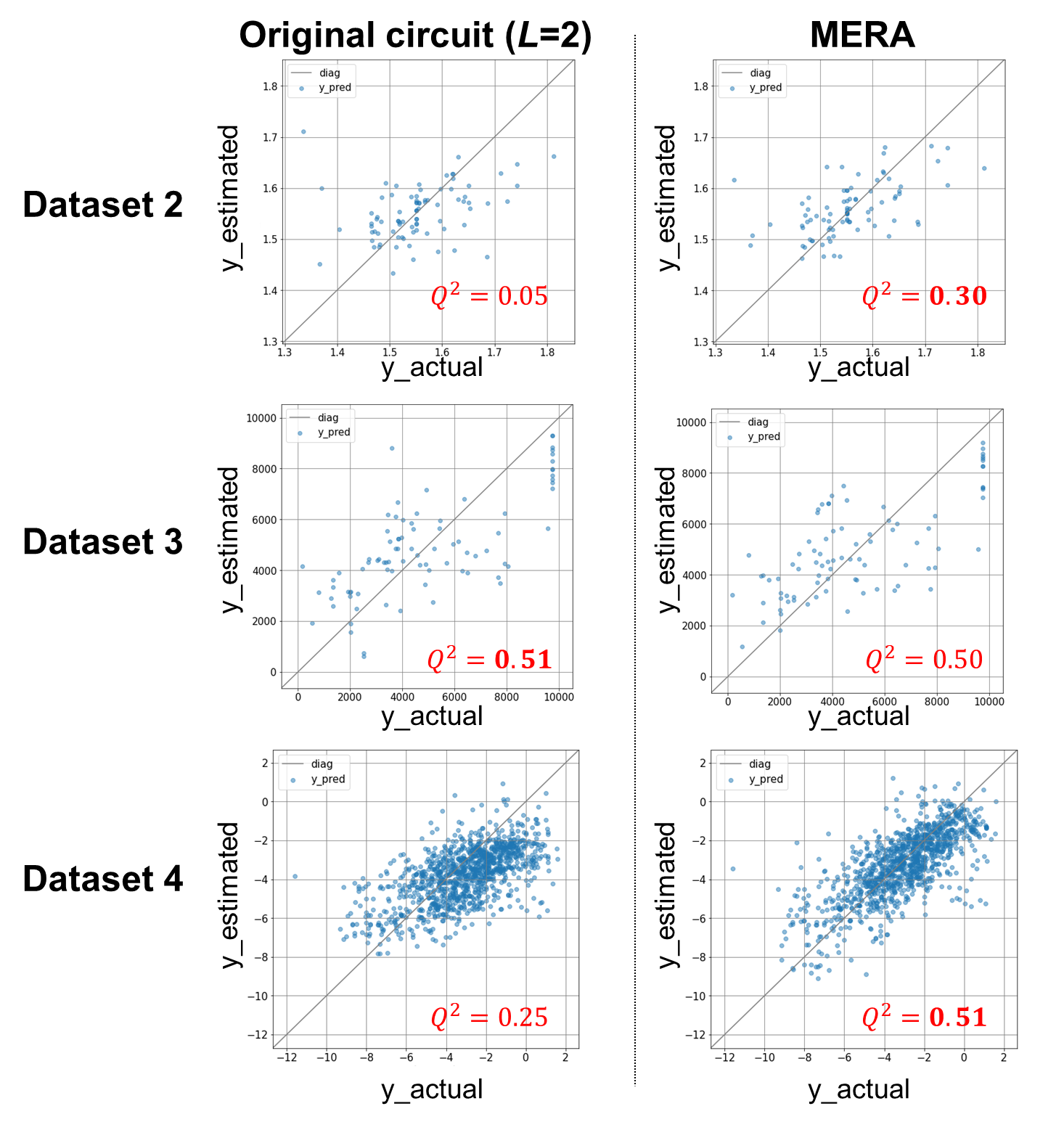


**Training the original circuit (L=2) using SGD** **combined with parameter-shift rule**

Figure S2. Dependence of the learning curve on the number of shots when the original circuit (L=2) is trained using SGD combined with the parameter-shift rule. We considered 100, 1000, and 10000 as the number of shots n to calculate each expected value. The initial values of the circuit parameters are the same as the case of Figure 4 in the main text.


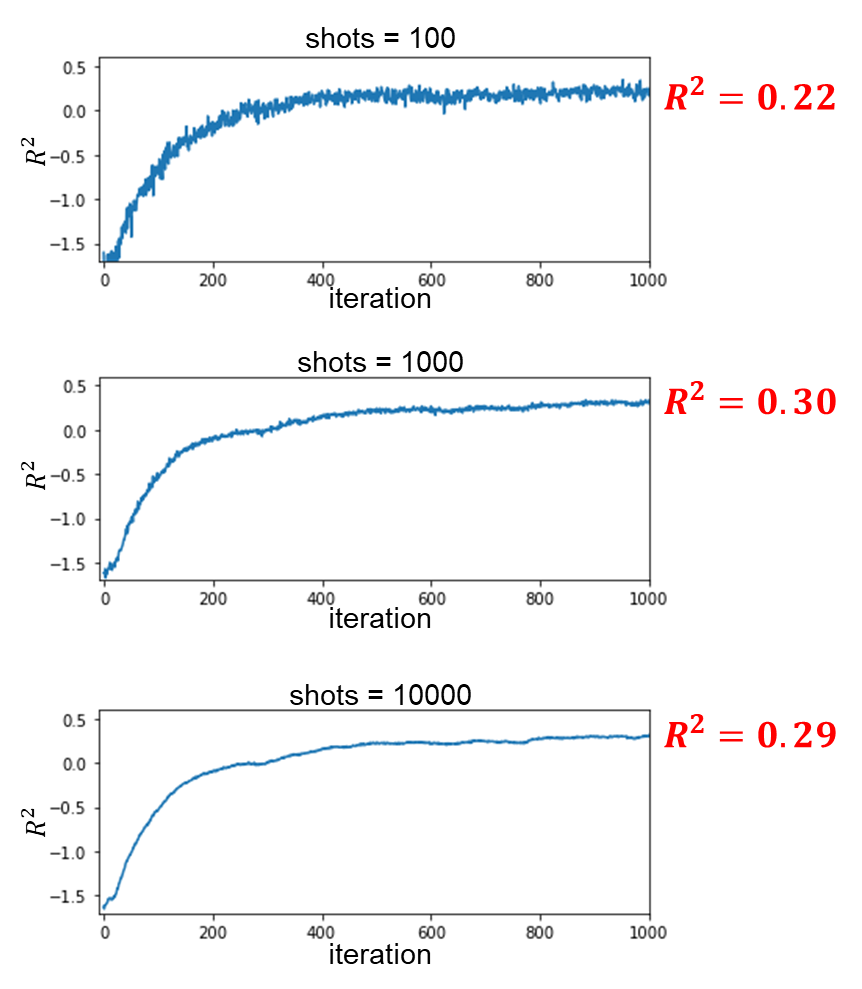

Supplement: Supplementary file 1 — Supplementary Information. [file 41598_2022_22940_MOESM1_ESM.docx]
